# Physiological and Psychological Effects of Watching Videos of Different Durations Showing Urban Bamboo Forests with Varied Structures

**DOI:** 10.3390/ijerph17103434

**Published:** 2020-05-14

**Authors:** Yuqian Wang, Mingyan Jiang, Yinshu Huang, Zhiyi Sheng, Xiao Huang, Wei Lin, Qibing Chen, Xi Li, Zhenghua Luo, Bingyang Lv

**Affiliations:** College of Landscape Architecture, Sichuan Agricultural University, Chengdu 611130, China; shewangyq@126.com (Y.W.); yezhukuaipao@163.com (Y.H.); s844475311@126.com (Z.S.); s20177605@163.com (X.H.); landscape1990@163.com (W.L.); cqb@sicau.edu.cn (Q.C.); lixi@sicau.edu.cn (X.L.); YLS@sicau.edu.cn (Z.L.); beyonglv@163.com (B.L.)

**Keywords:** natural therapy, bamboo forests, stimulation duration, forests structures, physiological and psychological responses, stress restoration

## Abstract

This study illustrated the physiological and psychological effects of watching videos of different durations showing bamboo forests with varied structures. Physiological indicators, including EEG (electroencephalogram), blood pressure, skin conductance, and pulse, were monitored in 180 Chinese university students (mean age: 20.72 ± 2.56 years) while they were watching bamboo videos. Before and after watching the videos, their psychological indicators, including positive and negative moods, were measured using the Profile of Mood States questionnaire. After watching the bamboo videos of different durations, all of the physiological indicators responded to the stimulation after only 1 min. The indicators showed different trends at 1, 3 and 5 min. EEG decreased and then was maintained at a stable level after 1 min, and the high β, low β, and α waves had no significant differences between 1, 3 and 5 min. Blood pressure dropped to a stable state after 3 min, and the decline was significantly different greater after 3 min than after 1 min. Skin conductance increased for 1 to 5 min, and it did not stabilize after a long time (5 min). Pulse decreased after 1 min but increased after 5 min. After watching the videos with bamboo of varying structures, the physiological and psychological indicators showed significantly different changes. Skin conductance significantly increased (mean value: 6.78%), and the amount of sweat was more effectively reduced, thereby reducing tension, when the students viewed videos of sympodial bamboo forests compared with monopodial bamboo forests. Bamboo forests with a higher canopy density (0.83–0.85) could significantly decrease α waves (mean value: 1.50 Hz), relaxing the human body. High β and low β waves showed greater decreases, with tension reduced more effectively, when bamboo forests with a low tilt ratio (< 1.5%) were viewed. Bamboo forests with neat undergrowth could have more beneficial physiological and psychological effects on the human body.

## 1. Introduction

With the development of global urbanization and increasing life pressures, modern people are affected by many negative forces, leading to an increase in physical and mental health issues [1,2].

To reduce the negative impact of the urban environment on people, an increasing number of studies have begun to attend to the benefits of the natural environment. After the term “forest bathing” was proposed in Japan in 1982, a large number of studies have shown that it is an effective practice for relieving stress [3,4,5,6]. Several studies have shown that time spent in a forest can decrease blood pressure (BP), the pulse rate [7], and the activity of the sympathetic nervous system [8], while also improving the human immune system [9,10]. Furthermore, the forest environment is more restorative than urban environments [11], and it can decrease anxiety, tension [12], and depression [13] while improving concentration [14]. Compared with natural forests, urban forests are easier for city dwellers to visit. Thus, constructing urban forests with different landscapes can provide more convenient forest bathing places for urban residents, effectively reducing their stress levels.

China, a country known as the “World’s Bamboo Kingdom”, has rich bamboo resources. Bamboo forests cover an area of 14 million acres in China, and in Sichuan Province alone, they cover 2.8 million acres, ranking it the top in the country [15]. As an important part of the forest ecosystem, bamboo forests have the ecological function of protecting the environment by conserving soil and water [16,17]. In addition, bamboo forests are commonly used for urban landscape greening because of the supply-side structural reform of forestry of China [18]. Bamboo is an important urban greening vegetation in China, which often used as the base plant for urban parks. By greening the urban environment with bamboo forests, health benefits can be effectively enjoyed by the local population [19]. However, previous studies have primarily focused on cypresses [20], oaks [21], pines [22], and other tree species; limited studies have been conducted on the health benefits of urban bamboo forests [23,24].

In the forest environment, different landscape elements, such as tree height, diameter at breast height (DBH), canopy density, and color can have different physiological and psychological effects on humans [25,26]. Similar to the forest environment [27], urban bamboo forests are also determined by many different elements including bamboo height, density, tilt, and undergrowth uniformity. However, no studies have discussed how these elements affect humans.

A large number of field studies have shown that direct forest stimuli impart physiological and psychological effects on humans. The methods used in these studies can effectively find the impact of beneficial substances in the forest on humans [28,29], but they are difficult, time-consuming, and easily affected by season, temperature, and weather. With the rapid development of image projection technology, many studies have confirmed that the beneficial effects of forests on humans are still present when using displayed images [30]. Some studies have also compared the benefits of direct and indirect nature stimuli on humans and found no significant difference between them [31]. As an emerging method of image research, videos can show landscapes more realistically and comprehensively in a three-dimensional space. However, no study has yet discussed whether videos of different durations have varying influences on humans.

Therefore, this study examined the different effects of bamboo forest videos of various durations and different landscape structures on humans. University students viewed videos of bamboo forests, and their physiological and psychological indicators were monitored.

## 2. Materials and Methods

### 2.1. Urban Bamboo Forests Sites

**Figure 1:**
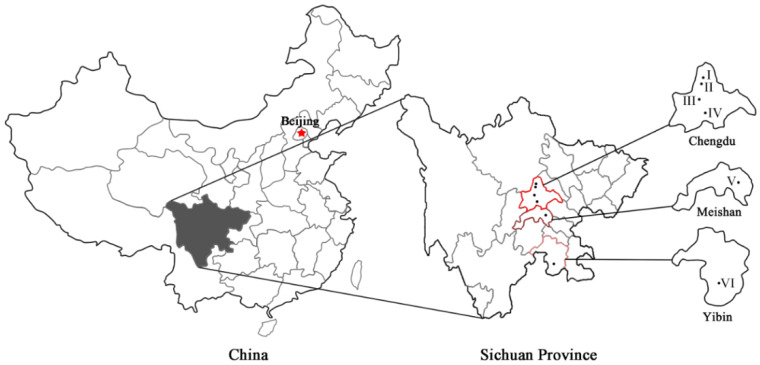
Maps of the six bamboo forest sites in this study. I—*Phyllostachys praecox* f. prevernalis in Zhuhaidongtian in Chengdu; II—*Phyllostachys praecox* f. prevernalis in Tongmagou in Chengdu; III—*Neosinocalamus affinis* in Chongzhou in Chengdu; IV—*Neosinocalamus affinis* in Xinjin in Chengdu; V—*Phyllostachys heterocycle* var. pubescens in Renshou in Meishan; VI—*Dendrocalamus latiflorus* in Changning in Yibin.

Six bamboo forest sites selected from three cities in China (Figure 1), four of which were located in Chengdu, Sichuan Province. The bamboo species were *Phyllostachys praecox* C. D. Chu et C. S. Chao f. prevernalis S. Y. Chen et C. Y. Yao (monopodial bamboo; two sites) and *Neosinocalamus affinis* (Rendle) Keng f. (sympodial bamboo; two sites), which are the dominant bamboo species in central Sichuan. The remaining two forests were located in southern Sichuan. One of the remaining two was located in Meishan, and the bamboo species was *Phyllostachys heterocycla (Carr.)* Mitford var. pubescens (Mazel ex H. de Lehaie) Ohwi (monopodial bamboo; one site). The other one was *Dendrocalamus latiflorus* Munro (sympodial bamboo; one site), situated in Yibin. Due to the higher temperatures and greater rainfall, the dominant bamboo in southern Sichuan is large.

The six bamboo forests all individually covered an area of more than 20 hectares. Three 20 m × 20 m quadrats were constructed in each bamboo forest to investigate the bamboo forest structures including bamboo species, bamboo height, DBH, density, canopy density, tilt ratio, and undergrowth uniformity (Table 1). The tilt ratio was calculated as xb/xa, where xa is the total number of bamboo individuals and xb is the number of bamboos with an inclination angle greater than 45°.

**Figure 2:**
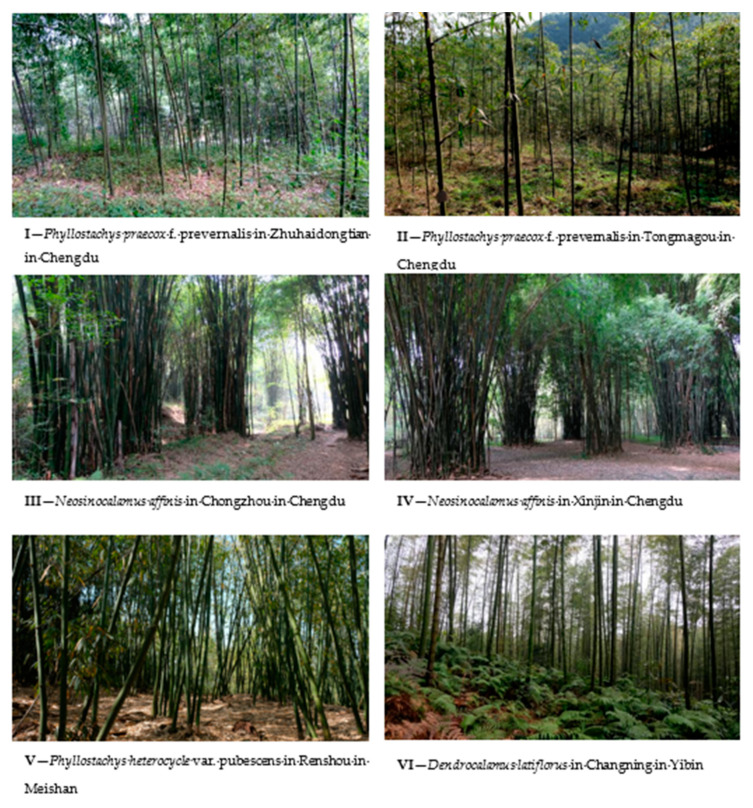
Urban bamboo forest sites.

The videos were prepared in the summer of 2018 from July to October. The same person used the same camera (XT-20, Fujifilm (China) Investment Co., Ltd., Shanghai, China) to film the urban bamboo forest videos in the above six bamboo forests (Figure 1 and Figure 2; Table 1).

### 2.2. Horizontal 360° Urban Bamboo Forest Videos

In each bamboo forest, the videos were filmed in the middle of the forest, more than 50 m away from the edge of the bamboo forest. Starting from the north direction, the shooting height was 1.5 m, rotated 360° clockwise, and the lens was constantly moving at a speed of 1.2°/s (Figure 2). In addition, filming was conducted in areas without water, roads, and facilities. Then, a video for each bamboo forest for the landscape stimulation experiment was produced. The final video length was 6 min (including 1 min of blank video (white screen) and 5 min of horizontal 360° urban bamboo forest footage).

### 2.3. Participants

One hundred and eighty Chinese university students ranging in age from 18 to 28 years (mean age: 20.72 ± 2.56 years; 90 males and 90 females) were randomly recruited from a university. To ensure that they understood the experiment, each subject was explained the details of forest bathing. All of the participants were without physical or mental illness. Smokers and alcoholics were also excluded. Alcohol consumption and vigorous physical activity were not allowed during the study period.

All of the subjects were randomly divided into six groups consisting of thirty individuals each (15 males and 15 females). The No.1 to No.6 groups were assigned to watch the No.1 to No.6 videos, respectively. Considering substantial previous research had proved the beneficial effects of direct and indirect contact with forests on the human body [24,25,26,31,32], no control group was set up in this study. Each participant was given a schedule for the experiment and was asked to be in the experimental room on time. Prior to the experiment, a full explanation of the research purpose and the experimental procedure was given to participants to prevent nervousness. Written informed consent was obtained from each subject. The study was performed with the approval of the local university Ethics Committee.

### 2.4. Experimental Sites

We chose 18 days in November 2018 (from 9:00 to 11:00 and 14:00 to 17:00 every experiment day) as experiment times. Three days were needed to finish the experiment for one group, and 10 participants were measured each day. The experiment was conducted in a chamber in the fourth teaching building of a local university. The waiting room and the experimental room were quiet, ventilated, and maintained at 19 to 24 °C, with a relative humidity of 40% to 50%, the conditions in which people feel most comfortable.

### 2.5. Experimental Design

Participants performed the experiment individually, and the duration of each experiment was 30 min. Before the experiment began, every participant was asked to fill in their basic personal information and received a description of the experimental procedure in the waiting room. Then, everyone was required to rest in their seats. Besides watching different bamboo forest videos in the six groups, all experimental procedures were the same for each participant. Each subject only watched one video; thus, there were no order effects.

**Figure 3:**
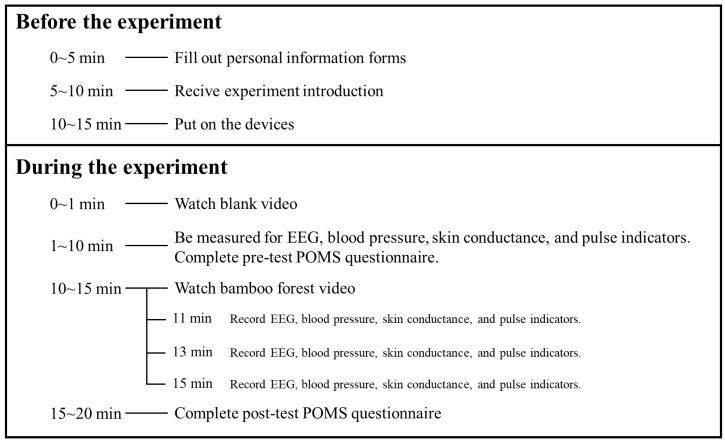
Experimental schedule for each participant. POMS, Profile of Mood States.

**Figure 4:**
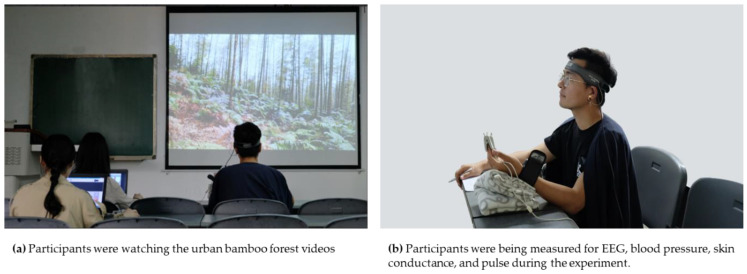
Participants in the experiment, (**a**) Participants were watching the urban bamboo forest videos; (**b**) Participants were being measured for EEG, blood pressure, skin conductance, and pulse during the experiment.

After devices for physiological measurement were fitted, each participant was sent to the experimental room individually. The experimental schedule is shown in Figure 3. Participants watched videos in sitting position (Figure 4). After watching the blank video (rest period), the participants were measured for EEG (electroencephalogram), skin conductance, blood pressure, and pulse and completed the pre-test POMS (Profile of Mood States) questionnaire. Then, the participants watched the bamboo video and their EEG, skin conductance, blood pressure, and pulse were continually measured. Physiological indicators were recorded when the participants watched the bamboo videos after 1, 3, and 5 min. After watching the bamboo forest videos, the participants completed the same POMS questionnaire again (Figure 4).

### 2.6. Data Collection

EEG (high β, low β and α wave (HZ)) results were recorded using Neuro Harmony EEG biofeedback technology. EEG is a change in the electrical waves when the brain is active. It is the reflection of the electrophysiological activity of the brain’s nerve cells on the surface of the cerebral cortex or scalp. α waves and β waves can reflect the emotional changes in people after being stimulated by the outside world. High β appears when the human body is nervous, and low β waves often appear when people are thinking. β waves increase when the mind is nervous, anxious, and thinking about problems, and the α waves decrease or disappear when the mind is concentrated and relaxed [32,33].

Blood pressure (systolic (mmHg), diastolic (mmHg), and pulse (bmp)) was measured using a sphygmomanometer (Omron, HEM-6322T, Omron, Tokyo, Japan). Changes in psychological stress and mood can cause changes in cardiovascular activity, which in turn affects pulse and blood pressure. Blood pressure includes systolic and diastolic blood pressure, which are commonly used to reflect the activity of the human sympathetic nervous system (SNS) and parasympathetic nervous system (PNS) [34,35]. In general, there will be an increase in blood output, a more rapid heartbeat, and an increase in systolic blood pressure when people are nervous, and nervousness also affects the peripheral resistance of the blood circulation, so peripheral resistance increases and diastolic blood pressure rises [36]. Pulse refers to the beating of the arteries caused by the shock of blood output when the human heart contracts. When the body is in motion or during emotional excitement, the pulse rate can increase, while rest and relaxation will slow the pulse rate [37].

Skin conductance was measured using a skin conductance sensor, HKR-11 C (Hefei Huake Electronic Technology Research and Production, Hefei, China). Because skin conductance varies from person to person, this experiment used the rate of change to analyze the skin conductance. This was calculated as follows: (xb−xa)/xa, where xa is the change value with the blank video and xb is the change value with the bamboo forest video. When people are emotionally stimulated, such as having nervousness and/or anxiety, the sympathetic nervous system causes the skin’s vertical muscles to contract, sweat secretion by the sweat glands increases, and the longitudinal resistance of the dermis decreases, which results in a decrease in human skin conductance. Measuring skin conductance can monitor the emotional state of the human body [38,39].

The average changes in the EEG, blood pressure, skin conductance, and pulse after 1, 3, and 5 min were calculated to represent the test values at 1, 3, and 5 min.

A shortened Chinese version of the POMS scale was used to evaluate psychological responses to videos of urban bamboo forests [40,41]. The POMS scale included 40 adjectives that could be consolidated into the following six effective dimensions: T (tension), D (depression), A (anger), S (self-esteem), F (fatigue), C (confusion), and V (vigor). Negative POMS scores (T+D+A+F+C) and positive POMS scores (H+S) were used to reflect human emotional changes.

### 2.7. Data Analysis

Excel 2016 and SPSS 20.0 (IBM Corp, Armonk, NY, USA) were used for statistical analyses. An analysis of variance (ANOVA) was used to compare the changes in physiological and psychological indicators after watching the bamboo videos with different durations and structures to investigate the significance levels. Pearson’s correlation test was used to analyze the correlation between bamboo structure indices and psychophysiological indices.

## 3. Results

### 3.1. Physiological Effects of Urban Bamboo Forest Videos of Different Durations on Humans

#### 3.1.1. EEG

**Figure 5:**
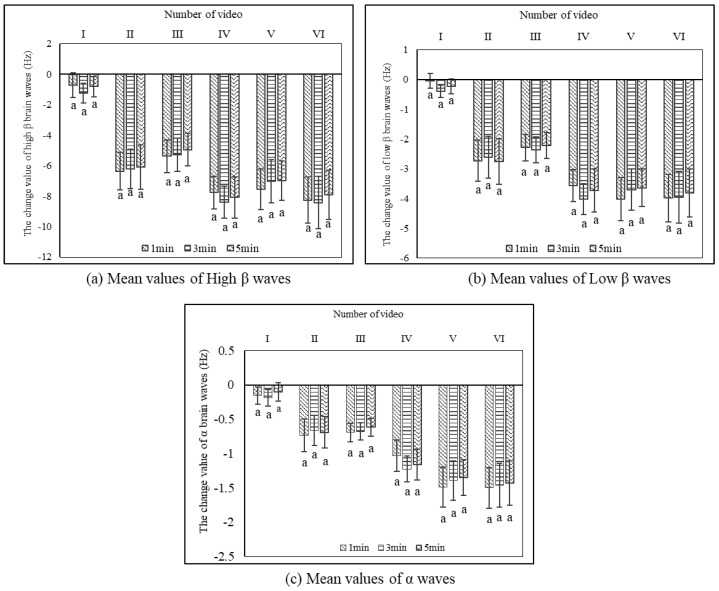
Changes in EEG mean values after watching the urban bamboo forest videos of different durations (*n* = 30; mean ± standard error). The same lowercase letter “a” represents no significant differences between 1, 3, and 5 min with each video for the EEG indicators. The zero point represents the value measured after participants watched the blank video.

After the participants watched the videos of urban bamboo forests, there was no significant difference in the changes of high β, low β, and α waves between the six videos with different durations (*p* > 0.05) (Figure 5). Each brain wave reached stability within 1 min; at this time, the mean value for the high β wave was −6.01 Hz; the low β wave, −2.77 Hz; and the α wave, −0.93 Hz. It indicated that EEG responded very fast to the videos of urban bamboo forests and could decrease to a stable state within a short time (1 min). Moreover, the values remained stable within 1 to 5 min.

#### 3.1.2. Blood Pressure

**Figure 6:**
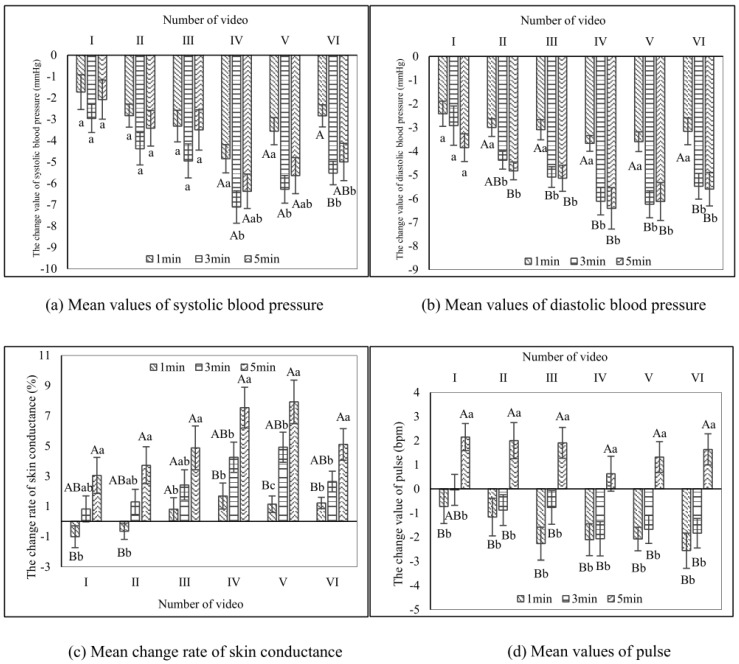
Changes in blood pressure, skin conductance, and pulse mean values after watching the urban bamboo forest videos of different durations (*n* = 30; mean ± standard error). Different capital letters represent extremely significant differences (*p* < 0.01) between 1, 3, and 5 min in each site for the blood pressure, skin conductance, and pulse indicators. Different lowercase letters represent significant differences (*p* < 0.05) between 1, 3, and 5 min in each site for the blood pressure, skin conductance, and pulse indicators. The same letters indicate no significant differences between 1, 3, and 5 min in each video. The zero point represents the value measured after participants watched the blank video.

After watching the videos of the urban bamboo forest, the blood pressure of viewers decreased after all six videos within 1 to 3 min (Figure 6). This decrease was significant (*p* < 0.05) in viewers watching most of the videos of bamboo forests (except Video I and Video II). Systolic blood pressure showed more of a decrease within 3 min, and the mean value was −5.20 mmHg, while diastolic blood pressure decreased by 5.04 mmHg. There was no significant difference in blood pressure changes between the six videos (*p* > 0.05) within 3 to 5 min. These results indicated that blood pressure responded quickly to the videos of urban bamboo forests. Moreover, blood pressure was stable up to 3 min and remained stable between 3 and 5 min.

#### 3.1.3. Skin Conductance

After the participants watched the videos of the urban bamboo forests, the rate of skin conductance increased continually over time for all of the six videos (Figure 6). Except for viewers watching Video III, the difference in the skin conductance of the viewers was extremely significantly (*p* < 0.01) higher at 5 min than at 1 min. These results showed that the skin conductance was continually affected by the stimulation and responded slowly to the videos of urban bamboo forests. Additionally, it did not stabilize after 5 min and may have continued to increase after the measurements conducted in this study concluded.

#### 3.1.4. Pulse

After watching the videos of the urban bamboo forests, the pulse of the viewers decreased at the beginning of the experiment, but as time went on, the pulse gradually recovered and continued to increase for all six videos (Figure 6). The pulse had the greatest decrease at 1 min, and it was not significantly different from at 3 min (*p* > 0.05). At 5 min, the pulse showed an increase, and the pulse was extremely significantly higher at 5 min than 3 min (*p* < 0.01) with an average of 1.61 bpm for most of the videos (except video I). These results indicated that the videos of the urban bamboo forest had an extremely rapid influence on the pulse, and the pulse responded to the stimulation after a short time (1 min). However, the pulse subsequently started to increase.

### 3.2. Physiological and Psychological Effects of Urban Bamboo Forest Videos Showing Different Structures

#### 3.2.1. Bamboo Species

There are many bamboo species, but only large and medium-sized bamboo species are suitable for building urban bamboo forests. Due to the different types of bamboo rhizome, monopodial bamboo and sympodial bamboo have different arrangements of the bamboo stalks on the ground.

All of the different video stimulations can increase human skin conductance. The average rate of increase was 5.37% when the video was watched for 5 min. The influence of the sympodial bamboo forests (mean value: 6.78%) on skin conductance was better than that of the monopodial bamboo forests. The increase in skin conductance from the medium-sized sympodial bamboo forests (Videos III and IV) could be 2.82% more than that achieved by medium-sized monopodial bamboo forests (Videos I and II). The increase in skin conductance observed in participants watching Video IV (7.54%) was significantly higher than for Video I (3.05%; *p* < 0.05). Additionally, the increase in skin conductance for large sympodial bamboo forests (Video V) could be 2.82% greater than that measured for large monopodial bamboo forests (Video VI). However, there was no significant difference between Video V (7.92%) and Video VI (5.10%) (Table 2). These results indicated that bamboo stalks gathered in clusters and with more open space at the ground level of sympodial bamboo forests can effectively reduce sweat secretion from the human body, thus reducing tension and anxiety.

#### 3.2.2. Bamboo Height, DBH, Density, and Canopy Density

Canopy density is an important index reflecting the structure and density of forests, which is affected by bamboo height, DBH, and density [42]. As shown in Table 3, no significantly positive correlations were observed between density and α waves (0.783, *p* > 0.05). Moreover, significant negative correlations were found between bamboo height and α waves (−0.870*, −0.837*, *p* < 0.05), and extremely significant negative correlations were found between canopy density and α waves (−0.938**, *p* < 0.01).

All of the video stimulations of different types of bamboo forest can decrease the α waves, and the mean decrease was 0.93 Hz while the video stimulation time was 1 min. Compared with medium-sized bamboo forests with small canopy density (Videos I, II, III, and IV), the mean α waves were lower for large bamboo forests with a higher canopy density (0.83–0.85; Videos V and VI). The mean decrease in the α waves of viewers watching Videos V and VI (mean value: 1.50 Hz) was significantly higher (*p* < 0.05) than that of viewers watching Video II (−0.73 Hz) and Video III (−0.69 Hz). Additionally, it was extremely significantly higher than in those watching Video I (−0.15 Hz) (Table 2). The results showed that although the density of large bamboo forests is relatively low, tall bamboo with a large DBH increased the canopy density. As the light intensity under the forest weakened, the forest was quieter, which can promote a decrease in α waves and make the brain more concentrated while relaxing.

#### 3.2.3. Tilt Ratio

The tilt ratio can be used to describe the sense of order in the interior space of urban bamboo forests [43]. Fallen bamboo impacts the stability and balance of bamboo forests. As shown in Table 3, no significantly negative correlations were observed between the tilt ratio and high β and low β waves (0.719, 0.730, *p* > 0.05). The six videos of bamboo forests can decrease the high β and low β waves, and the mean decrease was 6.01 and 2.77 Hz, respectively, after 1 min. Compared with bamboo forests with a high tilt ratio (Videos I and III), the mean high β and low β waves were both lower for the bamboo forests with low tilt ratios (<1.5%; Videos II, IV, V, and VI). The mean decreases in high β and low β waves in participants watching Video I (high β: −0.73 Hz; low β: −0.04 Hz) were extremely significantly lower than in those watching Videos II, IV, V, and VI (*p* < 0.01) (Table 2). These results indicate that a low tilt ratio makes the urban bamboo forest space neater, improving the visibility, which is conducive to a decrease in β waves and tension relief.

#### 3.2.4. Undergrowth Uniformity

Undergrowth uniformity can be used to describe the sense of order of the surface landscape of urban bamboo forests; this is affected by the consistency of height and the uniformity of coverage. As presented in Table 3, the undergrowth uniformity had significant negative relationships with high β waves, low β waves, and pulse (−0.817*, −0.842*, and 0.852*, respectively, *p* < 0.05). It also had extremely significant negative correlations with systolic blood pressure, diastolic blood pressure, and negative POMS scores (−0.951**, −0.940**, −0.923**, *p* < 0.01). Significant positive relationships were observed between undergrowth uniformity and positive POMS scores (0.882*, *p* < 0.01), and extremely significant positive correlations were observed with skin conductance (0.944**, *p* < 0.01). The results showed that neat undergrowth can not only clarify the vertical structure of the bamboo forests but also create more beautiful landscapes.

## 4. Discussion

### 4.1. Human Physiological Indices in Response to Urban Bamboo Forest Videos of Different Durations

Previous studies have shown that contact with nature directly or indirectly has benefits to human health [44,45]. Many physiological indices including EEG, blood pressure, pulse, salivary cortisol, and HRV (heart rate variability) were measured to evaluate the impact of the forest landscape on the human body. However, the trends of indices in different studies have different results, which is most likely due to the different durations of experimental time and the durations of time for which the indices were monitored. For example, this study indicates that compared with blood pressure, pulse, and other indicators, the process by which the sympathetic nervous system responded to the stimulation, causing sweat secretion to decrease, needed more time. Song et al. found that all mean heart rate values within 1-min epochs were lower during a walk in an urban park compared with those during a walk through a city, but significant levels were not reached until after 15 min [46]. This previous study showed that the difference in the duration of environmental stimulation influenced physiological indices. Ahmad et al. observed a significant difference in high β waves between city areas and bamboo forests after 1 and 5 min over the course of a 15-min experiment of walking through a bamboo forest, which is consistent with the findings in this study [33]. These results in this study indicate that high β, low β, and α waves can decrease significantly in a short time (1 min) regardless of whether the experiment was a field stimulation or video stimulation and that the physiological response remains stable for a long time (5 min).

Furthermore, several field experiments have reported that forest environments can also decrease blood pressure, but the level of significance was different in different studies [9,10,37,47,48]. Different study durations might also explain the variations reported in the decrease in blood pressure. For example, Lee presented twelve different landscape images of cities and gardens continuously for 90 s and observed significant differences in the diastolic blood pressure between participants viewing city and garden images but no significant difference in the systolic blood pressure [49]. Jeon et al. reported that no significant difference was found before and after experiences in the indirect nature group [31]. The results from these studies agree with our findings that systolic and diastolic blood pressure showed significant improvements after at least 3 min of video stimulation. The finding may explain the difference between the results of Lee and Jeon.

Moreover, some studies did not find any significant effects of forest on pulse [10,49], whereas some observed that it can significantly decrease pulse [50]. In this study, the pulse index began to increase after a brief decline, which was not observed in most previous studies. This result may be due to the environmental differences between the indoor and outdoor studies and the methods used for the video stimulation. Further research is needed on the changes in pulse indicators. Additionally, very few studies have reported the effects of different durations of urban forest bathing stimulation. In this study, an indoor experiment with four different types of physiological indicator was designed to determine the responses to 1 min, 3 min, and 5 min video stimulations, which can help with urban forest experiments in the future in terms of choosing a more appropriate experimental time. However, it is necessary to carry out further observations for a longer period of time in the future. In addition, more physiological indicators should be included, and studies and comparative studies of indoor and outdoor experiments should be conducted to confirm the results of this study.

### 4.2. The Different Physiological and Psychological Effects of Videos of Urban Bamboo Forests with Various Structures

Numerous studies on forest bathing have reported that forest environments with varying structures had positive impacts on humans compared with the urban environment; however, different structures of urban forest can have different effects on the human body [51]. Some studies have reported the differences in the physiological and psychological effects of different types of forest. Song et al. found that walking in urban parks during both fall and winter led to physiological and psychological relaxation effects [52,53], but more studies indicated the beneficial effects of forests in the spring [46,54]. Sonntag-Öström et al. considered that compared with autumn, spring improves wellbeing due to the elongated daylight hours in the spring [55]. Gerstenberg et al. argued that people can perceive the features of trees easily because of the full expansion of leaves in spring [56]. These findings correspond with the findings of this study, where the influence of the sympodial bamboo forests on the skin conductance was better than that of the monopodial bamboo forests, which mainly resulted from the concentrated and full look of the sympodial bamboo forests. Guan et al. reported the tree-species-specific effect of birch, maple, and oak trees on anxiety alleviation in university students [57]. Birch and oak were more effective in reducing anxiety regarding school work and social contact, which may be caused by the dark color in the maple forest and the white bark of birch trees. Additionally, the density of birch trees was lower than that of oak and maple trees, which may make the environment brighter. However, the results of the present study demonstrated that the higher the canopy density, the greater the decrease in α waves, allowing for a deeper relaxation. The differences in these results may be due to the influence of the cultural backgrounds of the participants. The traditional esthetics of bamboo forests in China tend to be elegant and quiet; further research is needed to confirm the findings of this study. This study also found that undergrowth uniformity could have beneficial effects on the physiological and psychological indices of subjects and better comprehensive benefits for the human body. Bamboo forests with more neat undergrowth are more beneficial to the human body. However, Lee et al. selected wild and tended forests in Seoul to compare the restorative effects of two different forests and found that the wild forest induced a significantly more favorable acute insulin reaction and better levels of oxidative stress than the tended forest [58]. These observations may be because the changes in the insulin index are inconsistent with those in other physiological indicators, or because viewing a large area of a single, neat landscape leads to a sense of monotony and fatigue, which adversely affects the human body [59]. Thus, the uniformity of a forest or bamboo forest may have a positive impact on humans within a certain range of area, which still needs to be further researched. In addition, bamboo forests are a special type of forest. Because of the thin stalks of bamboo, they are more prone to falling than other forests. This study found that the lower the tilt ratio, the more beneficial the physiological and psychological effects on the human body.

**Figure 7:**
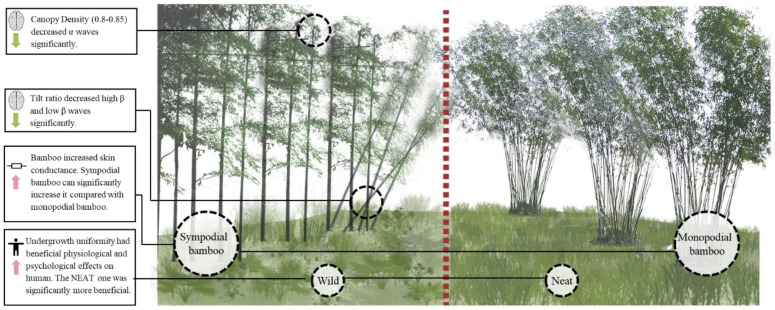
Psychophysiological effects of urban bamboo forest videos showing different structures.

Overall, bamboo is a special and important plant for urban greening. When constructing urban bamboo forests, it is possible to adopt directive breeding that selects large bamboo species and controls canopy density, tilt ratio, and undergrowth uniformity to enhance the benefits of the bamboo forest to the human body (Figure 7).

### 4.3. Limitations and Future Research

There are certain limitations to this study. First, the background of participants may have confounded the results. Information about whether the participants were from rural or urban areas and whether they had ever practiced forest bathing was unknown. Future research should evaluate the physiological and psychological responses according to the different background factors. Second, only 180 participants (30 per group) were recruited in the study. This sample size could not prove significant correlations between some indicators and landscape elements, and this might be caused by the relatively modest differences between various bamboo structures. To demonstrate the findings, further studies on a large sample are needed. Third, the study only used the landscape elements to qualitatively describe each bamboo forest instead of dividing the landscape elements separately for analysis or considering the interaction between each different landscape element. In the future, it will be necessary to further study the impact of individual landscape elements on the human body to confirm the results of this study. Lastly, we only measured mood to represent psychological indicators. Future research could employ eye-tracking technology to study the underlying psychological activities of participants.

## 5. Conclusions

The findings of this study illustrated that, when participants were stimulated by the landscape videos, the physiological indicators showed different trends at 1, 3 and 5 min. EEG decreased and then was maintained at a stable level after 1 min. The brain waves of people responded very quickly to the nature video stimulation, and the brain quickly became relaxed. Blood pressure dropped to a stable state after 3 min. Cardiovascular activity was affected quickly by the bamboo forest video but more slowly than the brain. The sympathetic nervous system was suppressed, and the parasympathetic nervous system was stimulated. Skin conductance increased for 1 to 5 min, and it did not stabilize after a long time (5 min). The bamboo forest video slowly reduced sweat secretion from the human body, thus reducing tension and anxiety. Pulse decreased after 1 min but increased after 5 min. After beginning to watch the videos, people were relaxed at first, but then they got nervous again.

Additionally, this study provided scientific evidence that watching videos with forests of varying structures leads to different beneficial effects on people. The forest with more open space at the ground level, a larger canopy density, a lower tilt ratio and neater undergrowth uniformity can make people feel more relaxed and comfortable, and it has more beneficial effects on the human body.

## Figures and Tables

**Table 1:** The characteristics of six bamboo forests sites.

Characteristics	Name					
	I—*Phyllostachys praecox* f. Prevernalis in Zhuhaidongtian in Chengdu	II—*Phyllostachys praecox* f. Prevernalis in Tongmagou in Chengdu	III—*Neosinocalamus affinis* in Chongzhou in Chengdu	IV—*Neosinocalamus affinis* in Xinjin in Chengdu	V—*Phyllostachys heterocycle* var. pubescens in Renshou in Meishan	VI—*Dendrocalamus latiflorus* in Changning in Yibin
Bamboo Species	Monopodial bamboo	Monopodial bamboo	Sympodial bamboo	Sympodial bamboo	Sympodial bamboo	Monopodial bamboo
Bamboo Height (m)	9.03 ± 0.47	8.63 ± 0.64	15.33 ± 0.75	14.97 ± 0.80	20.43 ± 0.85	18.67 ± 0.57
DBH (cm)	5.13 ± 0.31	5.20 ± 0.50	5.07 ± 0.47	5.17 ± 0.40	14.63 ± 1.07	12.33 ± 0.47
Density (strain/ha)	19,300 ± 1527	17,666 ± 577	22,000 ± 1732	21,300 ± 2000	5400 ± 360	3600 ± 360
Canopy Density	0.69 ± 0.02	0.71 ± 0.02	0.77 ± 0.04	0.78 ± 0.04	0.83 ± 0.08	0.85 ± 0.03
Tilt Ratio (%)	5.45 ± 1.99	0.35 ± 0.61	6.82 ± 0.62	1.27 ± 1.19	0.97 ± 0.87	0.62 ± 1.07
Undergrowth Uniformity	Wild	Normal	Normal	Neat	Neat	Normal

Note: Bamboo Species: according to different types of bamboo rhizomes; DBH (diameter at breast height): measuring the DBH of all bamboos in the quadrats and calculating the average value; Bamboo Height: choosing 5 mature bamboos with average DBH in the quadrats and measuring the height of bamboos from the base to the top after cutting; Density: number of bamboos per unit area in the quadrats; Canopy Density: the ratio of the total projected area of the canopy in the quadrat to the quadrat area; Tilt Ratio: The ratio of the number of bamboos with an inclination angle offset from vertical greater more than 45° with the total number of bamboos in the quadrats; Undergrowth Uniformity: according to the number, distribution, and height consistency of the undergrowth, dividing it into three levels: wild, normal, and neat.

**Table 2:** Changes in physiological and psychological indicators of each bamboo horizontal 360° video.

Physiological and Psychological Indicators	Video I	Video II	Video III	Video IV	Video V	Video VI
Skin Conductance (%)	3.05 ± 1.196 Ac	3.72 ± 1.23 Abc	4.88 ± 1.45 Aabc	7.54 ± 1.35 Aab	7.92 ± 1.44 Aa	5.10 ± 1.04 Aabc
α Wave (Hz)	−0.15 ± 0.13 Aa	−0.73 ± 0.24 ABab	−0.69 ± 0.13 ABab	−1.03 ± 0.23 ABbc	−1.49 ± 0.29 Bc	−22,121.5 ± 0.29 Bc
High β Wave (Hz)	−0.73 ± 0.82 Aa	−6.36 ± 1.25 Bb	−5.38 ± 1.07 Bb	−7.76 ± 1.08 Bb	−7.55 ± 1.34 Bb	−8.27 ± 1.52 Bb
Low β Wave (Hz)	−0.04 ± 0.25 Aa	−2.73 ± 0.70 Bb	−2.286 ± 0.44 Bb	−3.56 ± 0.55 Bb	−4.01 ± 0.73 Bb	−3.98 ± 0.79 Bb
Systolic Blood Pressure (mmHg)	−2.96 ± 0.66 Aa	−4.38 ± 0.76 ABab	−4.95 ± 0.80 ABab	−7.11 ± 0.76 Bc	−6.28 ± 0.65 Bbc	−5.52 ± 0.55 ABbc
Diastolic Blood Pressure (mmHg)	−2.92 ± 0.83 Aa	−4.38 ± 0.38 ABab	−5.09 ± 0.44 ABbc	−6.11 ± 0.58 Bbc	−6.24 ± 0.57 Bc	−5.48 ± 0.54 Bbc
Pulse (bmp)	−0.04 ± 0.64 a	−0.88 ± 0.64 a	−0.77 ± 0.70 a	−2.07 ± 0.71 a	−1.68 ± 0.58 a	−1.84 ± 0.61 a
Negative POMS Scores	−3.19 ± 0.49 Aa	−4.38 ± 0.59 Aab	−4.86 ± 1.30 Aab	−5.96 ± 0.93 Ab	−6.12 ± 0.68 Ab	−5.6 ± 0.83 Aab
Positive POMS Scores	−0.96 ± 0.671 a	0.17 ± 0.726 a	0.27 ± 0.519 a	0.59 ± 0.463 a	0.6 ± 0.440 a	0.48 ± 0.421 a

Note: The skin conductance used the mean value at 5 min; the high β, low β, and α waves used the mean value at 1 min; and the systolic blood pressure, diastolic blood pressure, and pulse used the mean value at 3 min. Different capital letters represent extremely significant differences (*p* < 0.01) between the six bamboo videos for the physiological and psychological indicators. Different lowercase letters represent significant differences (*p* < 0.05) between the six bamboo videos for the physiological and psychological indicators. The same letters represent no significant differences between the six videos.

**Table 3:** Pearson’s correlation coefficient between physiological and psychological indicators and the various landscape elements.

Physiological and Psychological Indicators	Bamboo Species	Density	Bamboo Height	DBH	Canopy Density	Tilt Ratio	Undergrowth Uniformity
Skin Conductance (%)	−0.780	−0.286	0.769	0.481	0.697	−0.429	0.944 **
α Waves (Hz)	0.289	0.783	−0.870 *	−0.837 *	−0.938 **	0.696	−0.732
High β Waves (Hz)	0.349	0.486	−0.675	−0.523	−0.783	0.719	−0.817 *
Low β Waves (Hz)	0.378	0.578	−0.756	−0.634	−0.840 *	0.730	−0.842 *
Systolic Blood Pressure (Mmhg)	0.685	0.246	−0.719	−0.384	−0.730	0.527	−0.951 **
Diastolic Blood Pressure (Mmhg)	0.685	0.383	−0.828*	−0.527	−0.831 *	0.507	−0.940 **
Pulse (Bmp)	0.413	0.476	−0.730	−0.540	−0.813 *	0.712	−0.852 *
Negative POMS Scores	0.617	0.466	−0.851 *	−0.595	−0.868 *	0.568	−0.923 **
Positive POMS Scores	−0.548	−0.370	0.719	0.462	0.775	−0.572	0.882 *

Note: DBH represents “diameter at breast height”; * *p* < 0.05; **p < 0.01.
